# 4-Amino­benzoic acid–4,4′-(propane-1,3-diyl)dipyridine (1/1)

**DOI:** 10.1107/S1600536810039528

**Published:** 2010-10-09

**Authors:** Fwu Ming Shen, Shie Fu Lush

**Affiliations:** a Department of Biotechnology, Yuanpei University, No. 306, Yuanpei St, HsinChu, 30015 Taiwan; b Department of General Education Center, Yuanpei University, No. 306, Yuanpei St, HsinChu, 30015 Taiwan

## Abstract

In the crystal structure of the title compound, C_13_H_14_N_2_·C_7_H_7_NO_2_, the 4,4′-trimethyl­ene-dipyridine (TMDP) mol­ecule displays an approximately planar structure, the maximum atomic deviation excluding H atoms being 0.118 (2) Å and the dihedral angle between the pyridine rings 4.59 (10)°. The TMDP and 4-amino­benzoic acid (ABA) mol­ecules are linked by O—H⋯N and N—H⋯N hydrogen bonding, while ABA mol­ecules are linked by O—H⋯O hydrogen bonding. C—H⋯π interactions are also observed between the methyl­ene groups of TMDP mol­ecules and the benzene rings of ABA mol­ecules.

## Related literature

For general background to 4-amino­benzoic acid as a ligand, see: Smith *et al.* (2005 ▶). For related structures, see: Lynch & McClenaghan (2001 ▶); Smith *et al.* (1997 ▶, 2000 ▶).
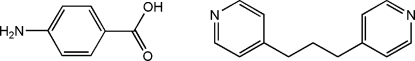


## Experimental

### 

#### Crystal data

C_13_H_14_N_2_·C_7_H_7_NO_2_
*M* *_r_* = 335.40
Monoclinic, 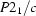

*a* = 7.6417 (6) Å
*b* = 11.1708 (9) Å
*c* = 20.8775 (18) Å
β = 99.436 (2)°
*V* = 1758.1 (2) Å^3^
*Z* = 4
Mo *K*α radiation
μ = 0.08 mm^−1^
*T* = 297 K
0.60 × 0.20 × 0.17 mm

#### Data collection

Bruker SMART 1000 CCD diffractometer
9816 measured reflections
3470 independent reflections
2055 reflections with *I* > 2σ(*I*)
*R*_int_ = 0.034

#### Refinement

*R*[*F* ^2^ > 2σ(*F* ^2^)] = 0.044
*wR*(*F* ^2^) = 0.131
*S* = 1.02
3470 reflections
238 parameters
3 restraints
H atoms treated by a mixture of independent and constrained refinement
Δρ_max_ = 0.14 e Å^−3^
Δρ_min_ = −0.18 e Å^−3^

### 

Data collection: *SMART* (Bruker, 2000 ▶); cell refinement: *SAINT* (Bruker, 1999 ▶); data reduction: *SAINT*; program(s) used to solve structure: *SHELXTL* (Sheldrick, 2008 ▶); program(s) used to refine structure: *SHELXTL*; molecular graphics: *ORTEP-3* (Farrugia, 1997 ▶); software used to prepare material for publication: *PLATON* (Spek, 2009 ▶).

## Supplementary Material

Crystal structure: contains datablocks global, I. DOI: 10.1107/S1600536810039528/xu5045sup1.cif

Structure factors: contains datablocks I. DOI: 10.1107/S1600536810039528/xu5045Isup2.hkl

Additional supplementary materials:  crystallographic information; 3D view; checkCIF report

## Figures and Tables

**Table 1:** Hydrogen-bond geometry (Å, °) *Cg*3 is the centroid of the C1–C6 ring.

*D*—H⋯*A*	*D*—H	H⋯*A*	*D*⋯*A*	*D*—H⋯*A*
O2—H2*A*⋯N2	0.82 (2)	1.81 (2)	2.632 (2)	179 (3)
N1—H1*A*⋯N3^i^	0.86 (2)	2.19 (2)	3.045 (3)	172 (2)
N1—H1*B*⋯O1^ii^	0.86 (1)	2.30 (1)	3.151 (3)	170 (1)
C13—H13*A*⋯*Cg*3^iii^	0.97	2.87	3.6606 (17)	139
C14—H14*A*⋯*Cg*3^iv^	0.97	2.88	3.6902 (17)	142

Symmetry codes: (i) 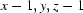; (ii) 
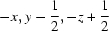
; (iii) 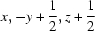; (iv) 
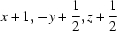
.

**Fig. 1.**
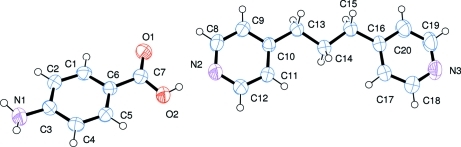
View of the title compound with the atom numbering scheme. Displacement ellipsoids for non-H atoms are drawn at the 50% probability level.

**Fig. 2.**
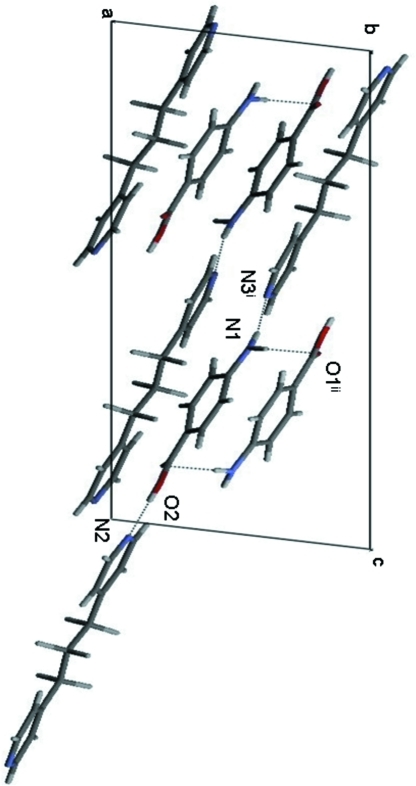
The molecular packing for the title compound, viewed along the b axis. Hydrogen bonds are shown as dashed lines.

## supplementary crystallographic information

### Comment

4-Aminobenzoic acid is a useful ligand for structure extension through both the carboxylic acid and amine functional groups, forming linear hydrogen bonding associations (Smith *et al.*, 2005). Other related reports with 4-aminobenzoic acid and Lewis base such as 4-(4-nitrobenzyl)pyridine (Smith, 1997), 4-aminobenzonitrile (smith *et al.*, 2000) and 2-amino-4-(4-pyridyl)pyrimidine (Lynch & McClenaghan, 2001).

We present here the crystal structure analysis of the 1:1 4-aminobenzoic acid and 4,4'-trimethylene-dipyridine adduct (Fig 1). In the title compound, C_13_H_14_N_2_.C_7_H_7_NO_2_, comprises one 4-aminobenzoic acid molecule and one 4,4'-trimethylene-dipyridine molecule, with no proton transfer. The dihedral angle between pyridyl rings for the molecule is 4.59 (10) °.

4-Aminobenzoic acid molecules are linked by O—H···N hydrogen bonds to 4,4'-trimethylene-dipyridine, forming linear hydrogen bonding. The structure exhibits a hydrogen-bonding network involving NH···N(prridyl) [N···N 3.043 (3) Å], amine and carboxylic N—H··· O [N···O 3.152 (3) Å] (Table 1 and Fig. 2), respectively.

This layer is consolidated by C—H···π stackings, the distance between C13—H13A^iii^···*Cg*3(C1—C6) and C14—H14A^iv^···*Cg*3 are 2.87 and 2.88 Å [symmetry code: (iii) = *X*,1/2-Y,1/2+*Z*; (iV) = 1+*X*, 1/2-Y, 1/2+*Z*].

### Experimental

The 4-aminobenzoic acid (137 mg, 1.0 mmol) and 4,4'-trimethylene-dipyridine (198 mg, 1.0 mmol) were dissolved in 20 ml methanol-water (1:1), the solution was refluxed for 30 min. The filtered solution was transferred to a 25 ml tube after one week at room temperature, and colorless transparent crystals formed (yield 50.22%).

### Refinement

Water H and amino H atoms were located in a difference Fourier map and were refined isotropically with the distance constraints of O—H = 0.820±0.001 and N—H = 0.860±0.001 Å. Other H atoms were positioned geometrically with C—H = 0.93 (aromatic) and 0.97 Å (methylene), and refined using a riding model with U_iso_(H) = 1.2U_eq_(C).

### Crystal data

**Table:**

C_13_H_14_N_2_·C_7_H_7_NO_2_	*F*(000) = 712
*M**_r_* = 335.40	*D*_x_ = 1.267 Mg m^−^^3^
Monoclinic, *P*2_1_/*c*	Mo *K*α radiation, λ = 0.71073 Å
Hall symbol: -P 2ybc	Cell parameters from 2559 reflections
*a* = 7.6417 (6) Å	θ = 2.7–25.5°
*b* = 11.1708 (9) Å	µ = 0.08 mm^−^^1^
*c* = 20.8775 (18) Å	*T* = 297 K
β = 99.436 (2)°	Block, colorless
*V* = 1758.1 (2) Å^3^	0.60 × 0.20 × 0.17 mm
*Z* = 4	

### Data collection

**Table:**

Bruker SMART 1000 CCD diffractometer	2055 reflections with *I* > 2σ(*I*)
Radiation source: fine-focus sealed tube	*R*_int_ = 0.034
graphite	θ_max_ = 26.0°, θ_min_ = 2.0°
φ and ω scans	*h* = −9→9
9816 measured reflections	*k* = −12→13
3470 independent reflections	*l* = −21→25

### Refinement

**Table:**

Refinement on *F*^2^	Primary atom site location: structure-invariant direct methods
Least-squares matrix: full	Secondary atom site location: difference Fourier map
*R*[*F*^2^ > 2σ(*F*^2^)] = 0.044	Hydrogen site location: inferred from neighbouring sites
*wR*(*F*^2^) = 0.131	H atoms treated by a mixture of independent and constrained refinement
*S* = 1.01	*w* = 1/[σ^2^(*F*_o_^2^) + (0.0618*P*)^2^ + 0.1063*P*] where *P* = (*F*_o_^2^ + 2*F*_c_^2^)/3
3470 reflections	(Δ/σ)_max_ < 0.001
238 parameters	Δρ_max_ = 0.14 e Å^−^^3^
3 restraints	Δρ_min_ = −0.18 e Å^−^^3^

### Special details

**Table:**

Geometry. All esds (except the esd in the dihedral angle between two l.s. planes) are estimated using the full covariance matrix. The cell esds are taken into account individually in the estimation of esds in distances, angles and torsion angles; correlations between esds in cell parameters are only used when they are defined by crystal symmetry. An approximate (isotropic) treatment of cell esds is used for estimating esds involving l.s. planes.
Refinement. Refinement of F^2^ against ALL reflections. The weighted R-factor wR and goodness of fit S are based on F^2^, conventional R-factors R are based on F, with F set to zero for negative F^2^. The threshold expression of F^2^ > 2sigma(F^2^) is used only for calculating R-factors(gt) etc. and is not relevant to the choice of reflections for refinement. R-factors based on F^2^ are statistically about twice as large as those based on F, and R- factors based on ALL data will be even larger.

### Fractional atomic coordinates and isotropic or equivalent isotropic displacement parameters (Å^2^)

**Table:**

	*x*	*y*	*z*	*U*_iso_*/*U*_eq_	
O1	0.2978 (2)	0.32540 (12)	0.38052 (6)	0.0690 (4)	
O2	0.3140 (2)	0.13738 (12)	0.41752 (7)	0.0628 (4)	
N1	−0.0339 (3)	0.04320 (17)	0.12447 (8)	0.0627 (5)	
C1	0.1876 (2)	0.24074 (15)	0.25210 (9)	0.0464 (5)	
H1	0.2285	0.3192	0.2564	0.056*	
C2	0.1117 (2)	0.19990 (15)	0.19194 (9)	0.0470 (5)	
H2	0.1027	0.2507	0.1563	0.056*	
C3	0.0479 (2)	0.08273 (15)	0.18373 (8)	0.0449 (4)	
C4	0.0743 (2)	0.00735 (15)	0.23811 (9)	0.0490 (5)	
H4	0.0405	−0.0726	0.2334	0.059*	
C5	0.1492 (2)	0.04932 (15)	0.29817 (8)	0.0450 (4)	
H5	0.1630	−0.0021	0.3337	0.054*	
C6	0.2049 (2)	0.16768 (15)	0.30680 (8)	0.0422 (4)	
C7	0.2759 (3)	0.21774 (17)	0.37126 (9)	0.0497 (5)	
N2	0.4334 (2)	0.23425 (14)	0.53122 (8)	0.0576 (4)	
N3	0.8995 (3)	0.22955 (18)	1.01679 (8)	0.0715 (5)	
C8	0.4148 (3)	0.35141 (18)	0.53916 (10)	0.0701 (6)	
H8	0.3645	0.3966	0.5034	0.084*	
C9	0.4660 (3)	0.40938 (18)	0.59730 (9)	0.0643 (6)	
H9	0.4518	0.4918	0.5999	0.077*	
C10	0.5383 (2)	0.34557 (15)	0.65190 (8)	0.0433 (4)	
C11	0.5601 (3)	0.22429 (16)	0.64341 (9)	0.0556 (5)	
H11	0.6105	0.1771	0.6783	0.067*	
C12	0.5072 (3)	0.17298 (18)	0.58325 (9)	0.0599 (6)	
H12	0.5241	0.0911	0.5789	0.072*	
C13	0.5864 (2)	0.40875 (15)	0.71617 (8)	0.0480 (5)	
H13A	0.4796	0.4454	0.7268	0.058*	
H13B	0.6680	0.4730	0.7107	0.058*	
C14	0.6693 (2)	0.33409 (16)	0.77385 (8)	0.0467 (5)	
H14A	0.7819	0.3024	0.7659	0.056*	
H14B	0.5922	0.2670	0.7791	0.056*	
C15	0.6987 (3)	0.40804 (16)	0.83572 (8)	0.0506 (5)	
H15A	0.7788	0.4730	0.8299	0.061*	
H15B	0.5862	0.4436	0.8410	0.061*	
C16	0.7721 (2)	0.34403 (17)	0.89766 (9)	0.0482 (5)	
C17	0.7995 (3)	0.22242 (19)	0.90228 (10)	0.0707 (6)	
H17	0.7763	0.1750	0.8653	0.085*	
C18	0.8616 (3)	0.1705 (2)	0.96176 (11)	0.0794 (7)	
H18	0.8776	0.0879	0.9630	0.095*	
C19	0.8731 (3)	0.3476 (2)	1.01235 (10)	0.0758 (7)	
H19	0.8981	0.3926	1.0502	0.091*	
C20	0.8115 (3)	0.4068 (2)	0.95554 (10)	0.0671 (6)	
H20	0.7961	0.4894	0.9559	0.080*	
H1A	−0.062 (3)	0.0975 (14)	0.0954 (8)	0.081 (8)*	
H1B	−0.094 (2)	−0.0219 (10)	0.1227 (10)	0.078 (8)*	
H2A	0.350 (3)	0.167 (2)	0.4531 (6)	0.103 (9)*	

### Atomic displacement parameters (Å^2^)

**Table:**

	*U*^11^	*U*^22^	*U*^33^	*U*^12^	*U*^13^	*U*^23^
O1	0.0985 (12)	0.0443 (8)	0.0564 (9)	−0.0035 (8)	−0.0104 (8)	−0.0094 (7)
O2	0.0919 (11)	0.0506 (8)	0.0408 (8)	0.0001 (7)	−0.0047 (7)	−0.0014 (7)
N1	0.0825 (14)	0.0561 (11)	0.0444 (10)	−0.0045 (11)	−0.0043 (9)	−0.0057 (10)
C1	0.0511 (11)	0.0361 (9)	0.0506 (11)	−0.0010 (8)	0.0043 (9)	−0.0006 (8)
C2	0.0559 (12)	0.0445 (10)	0.0398 (10)	0.0029 (9)	0.0052 (9)	0.0050 (8)
C3	0.0484 (11)	0.0443 (10)	0.0411 (10)	0.0031 (8)	0.0046 (8)	−0.0053 (8)
C4	0.0589 (12)	0.0366 (9)	0.0509 (11)	−0.0040 (9)	0.0068 (9)	−0.0032 (8)
C5	0.0511 (11)	0.0414 (10)	0.0418 (10)	0.0007 (8)	0.0052 (8)	0.0043 (8)
C6	0.0434 (10)	0.0399 (9)	0.0414 (10)	0.0032 (8)	0.0016 (8)	−0.0026 (8)
C7	0.0536 (12)	0.0464 (11)	0.0469 (11)	0.0037 (9)	0.0012 (9)	−0.0016 (9)
N2	0.0750 (12)	0.0542 (10)	0.0403 (9)	−0.0031 (9)	−0.0007 (8)	−0.0030 (8)
N3	0.0816 (13)	0.0886 (14)	0.0413 (10)	0.0064 (11)	0.0011 (9)	0.0041 (10)
C8	0.1005 (18)	0.0587 (13)	0.0430 (12)	0.0066 (12)	−0.0118 (11)	0.0034 (10)
C9	0.0930 (17)	0.0475 (11)	0.0469 (12)	0.0052 (11)	−0.0046 (11)	0.0028 (10)
C10	0.0459 (11)	0.0444 (10)	0.0388 (10)	−0.0034 (8)	0.0047 (8)	−0.0008 (8)
C11	0.0769 (14)	0.0484 (11)	0.0390 (11)	0.0047 (10)	0.0019 (10)	0.0027 (9)
C12	0.0829 (15)	0.0472 (11)	0.0473 (12)	−0.0006 (11)	0.0038 (11)	−0.0031 (9)
C13	0.0558 (12)	0.0454 (10)	0.0414 (10)	−0.0013 (9)	0.0042 (9)	−0.0026 (9)
C14	0.0506 (11)	0.0498 (10)	0.0390 (10)	0.0000 (9)	0.0050 (8)	−0.0014 (8)
C15	0.0573 (12)	0.0524 (11)	0.0411 (10)	−0.0008 (9)	0.0052 (9)	−0.0024 (9)
C16	0.0488 (11)	0.0576 (12)	0.0380 (10)	−0.0023 (9)	0.0062 (8)	−0.0029 (9)
C17	0.1055 (18)	0.0632 (13)	0.0407 (12)	0.0134 (13)	0.0039 (11)	−0.0054 (10)
C18	0.115 (2)	0.0710 (14)	0.0498 (14)	0.0199 (14)	0.0069 (13)	0.0050 (12)
C19	0.0960 (19)	0.0853 (17)	0.0408 (13)	−0.0093 (14)	−0.0048 (12)	−0.0106 (12)
C20	0.0840 (16)	0.0632 (13)	0.0495 (13)	−0.0069 (12)	−0.0026 (11)	−0.0086 (11)

### Geometric parameters (Å, °)

**Table:**

O1—C7	1.225 (2)	C9—H9	0.9300
O2—C7	1.316 (2)	C10—C11	1.380 (2)
O2—H2A	0.819 (15)	C10—C13	1.507 (2)
N1—C3	1.365 (2)	C11—C12	1.379 (3)
N1—H1A	0.860 (16)	C11—H11	0.9300
N1—H1B	0.858 (13)	C12—H12	0.9300
C1—C2	1.372 (2)	C13—C14	1.515 (2)
C1—C6	1.392 (2)	C13—H13A	0.9700
C1—H1	0.9300	C13—H13B	0.9700
C2—C3	1.398 (2)	C14—C15	1.519 (2)
C2—H2	0.9300	C14—H14A	0.9700
C3—C4	1.401 (2)	C14—H14B	0.9700
C4—C5	1.373 (2)	C15—C16	1.503 (2)
C4—H4	0.9300	C15—H15A	0.9700
C5—C6	1.392 (2)	C15—H15B	0.9700
C5—H5	0.9300	C16—C17	1.376 (3)
C6—C7	1.476 (2)	C16—C20	1.387 (3)
N2—C12	1.329 (2)	C17—C18	1.382 (3)
N2—C8	1.330 (2)	C17—H17	0.9300
N3—C18	1.316 (3)	C18—H18	0.9300
N3—C19	1.335 (3)	C19—C20	1.373 (3)
C8—C9	1.375 (3)	C19—H19	0.9300
C8—H8	0.9300	C20—H20	0.9300
C9—C10	1.381 (2)		
			
C7—O2—H2A	112.9 (18)	C10—C11—H11	120.0
C3—N1—H1A	115.9 (15)	N2—C12—C11	123.50 (18)
C3—N1—H1B	118.4 (15)	N2—C12—H12	118.2
H1A—N1—H1B	120 (2)	C11—C12—H12	118.2
C2—C1—C6	121.66 (16)	C10—C13—C14	117.31 (15)
C2—C1—H1	119.2	C10—C13—H13A	108.0
C6—C1—H1	119.2	C14—C13—H13A	108.0
C1—C2—C3	120.68 (17)	C10—C13—H13B	108.0
C1—C2—H2	119.7	C14—C13—H13B	108.0
C3—C2—H2	119.7	H13A—C13—H13B	107.2
N1—C3—C2	120.86 (17)	C13—C14—C15	111.15 (15)
N1—C3—C4	121.59 (17)	C13—C14—H14A	109.4
C2—C3—C4	117.54 (16)	C15—C14—H14A	109.4
C5—C4—C3	121.20 (16)	C13—C14—H14B	109.4
C5—C4—H4	119.4	C15—C14—H14B	109.4
C3—C4—H4	119.4	H14A—C14—H14B	108.0
C4—C5—C6	121.04 (16)	C16—C15—C14	117.08 (16)
C4—C5—H5	119.5	C16—C15—H15A	108.0
C6—C5—H5	119.5	C14—C15—H15A	108.0
C5—C6—C1	117.71 (16)	C16—C15—H15B	108.0
C5—C6—C7	122.47 (16)	C14—C15—H15B	108.0
C1—C6—C7	119.81 (16)	H15A—C15—H15B	107.3
O1—C7—O2	123.08 (17)	C17—C16—C20	115.43 (19)
O1—C7—C6	122.41 (17)	C17—C16—C15	124.14 (17)
O2—C7—C6	114.51 (16)	C20—C16—C15	120.40 (18)
C12—N2—C8	116.41 (17)	C16—C17—C18	120.2 (2)
C18—N3—C19	115.21 (19)	C16—C17—H17	119.9
N2—C8—C9	123.57 (19)	C18—C17—H17	119.9
N2—C8—H8	118.2	N3—C18—C17	124.6 (2)
C9—C8—H8	118.2	N3—C18—H18	117.7
C8—C9—C10	120.21 (18)	C17—C18—H18	117.7
C8—C9—H9	119.9	N3—C19—C20	124.3 (2)
C10—C9—H9	119.9	N3—C19—H19	117.9
C11—C10—C9	116.17 (17)	C20—C19—H19	117.9
C11—C10—C13	123.86 (17)	C19—C20—C16	120.3 (2)
C9—C10—C13	119.96 (16)	C19—C20—H20	119.9
C12—C11—C10	120.10 (18)	C16—C20—H20	119.9
C12—C11—H11	120.0		

### Hydrogen-bond geometry (Å, °)

**Table:**

Cg3 is the centroid of the C1–C6 ring.

**Table:**

*D*—H···*A*	*D*—H	H···*A*	*D*···*A*	*D*—H···*A*
O2—H2A···N2	0.82 (2)	1.81 (2)	2.632 (2)	179 (3)
N1—H1A···N3^i^	0.86 (2)	2.19 (2)	3.045 (3)	172 (2)
N1—H1B···O1^ii^	0.86 (1)	2.30 (1)	3.151 (3)	170 (1)
C13—H13A···Cg3^iii^	0.97	2.87	3.6606 (17)	139
C14—H14A···Cg3^iv^	0.97	2.88	3.6902 (17)	142

Symmetry codes: (i) *x*−1, *y*, *z*−1; (ii) −*x*, *y*−1/2, −*z*+1/2; (iii) *x*, −*y*+1/2, *z*+1/2; (iv) *x*+1, −*y*+1/2, *z*+1/2.
